# Early Right Heart Failure After Left Ventricular Assist Device Implantation: A Narrative Review of Definitions, Risk Prediction, and Management

**DOI:** 10.1155/cdr/2611216

**Published:** 2026-10-27

**Authors:** Qing Gao, Shujie Yan, Kunyu Li, Luyang Xu, Guodong Gao, Qiang Hu, Jinping Liu, Bingyang Ji

**Affiliations:** ^1^ Department of Cardiopulmonary Bypass Fuwai Hospital National Center for Cardiovascular Diseases Chinese Academy of Medical Sciences and Peking Union Medical College Beijing China pumc.edu.cn; ^2^ Pediatric Cardiac Center Fuwai Hospital National Center for Cardiovascular Diseases Chinese Academy of Medical Sciences and Peking Union Medical College Beijing China pumc.edu.cn

**Keywords:** extracorporeal membrane oxygenation, left ventricular assist device, mechanical circulatory support, right heart failure, right ventricle, risk prediction

## Abstract

Early right heart failure (RHF) remains common after left ventricular assist device (LVAD) implantation and is associated with substantial morbidity and mortality. Definition and time window determine reported incidence and complicate risk‐model comparison. Established risk models perform differently across cohorts and device eras, and temporary support should be matched to the dominant physiology. LV unloading alters venous return, septal geometry, and right ventricular (RV) loading, whereas perioperative stress exposes limited RV reserve. No single clinical, imaging, or hemodynamic measure reliably predicts early RHF, and existing scores perform variably on external validation. Dynamic hemodynamic assessment adds information about RV reserve and warrants prospective threshold validation. Management begins by addressing reversible causes and optimizing LVAD support. When temporary mechanical support is required, RV‐specific bypass restores pulmonary forward flow, an oxygenated right atrial‐to‐pulmonary arterial RVAD adds gas exchange, and venoarterial extracorporeal membrane oxygenation provides broader rescue for combined circulatory and respiratory failure. Comparative evidence on timing and configuration remains observational.

## 1. Introduction

Durable LVAD outcomes have improved substantially, but early right heart failure (RHF) remains common and clinically consequential [1, 2]. In a 2026 analysis of 2200 HeartMate 3 recipients from the MOMENTUM 3 portfolio using the 2020 MCS‐ARC criteria, 402 patients (18.3%) developed early RHF and 141 (6.4%) required RVAD support. Two‐year survival was 62.0% in patients with early RHF compared with 84.6% in those without it [3].

Early RHF is a heterogeneous syndrome produced by limited pre‐existing RV reserve and changes in loading conditions, ventricular geometry, and contractile demand during surgery and LV unloading. The biological heterogeneity shapes risk stratification and treatment selection, whereas inconsistent RHF definitions alter incidence estimates and risk‐model performance. Early RHF is a key period for considering temporary support, informed by preimplant risk and perioperative physiology.

The RHF definition determines which events are counted. Endpoints, populations, device eras, and center practice shape risk‐model transportability, whereas temporary support must match the dominant physiological problem. Contemporary clinical interpretation rests on definition‐specific incidence, model calibration in the target cohort, and physiology‐based support selection [4–6]. Device‐specific studies should be interpreted in the context of their design, endpoints, comparators, funding sources, and any relevant relationships between the authors and industry.

## 2. Literature Approach

This narrative review was informed by PubMed searches conducted on September 29, 2026, covering database inception to that date. Search terms combined left ventricular assist devices and RHF with concepts related to definitions and incidence, risk prediction, imaging and hemodynamics, tricuspid regurgitation, temporary right ventricular support, and extracorporeal membrane oxygenation; complete search strings and record counts are provided in Table S1. No language filter was applied, although all included studies were published in English. The review focused on adults receiving durable LVAD support. Consensus statements, systematic reviews, randomized trials, multicenter and external‐validation cohorts, and contemporary continuous‐flow LVAD studies were prioritized; smaller observational studies and case reports were considered when evidence was sparse or interventions were emerging. Reference lists of key publications were also examined. Pediatric‐only studies, RHF unrelated to durable LVAD support, duplicate reports without distinct outcomes, and studies outside the review scope were not retained. Selection was narrative rather than protocol‐driven. Evidence was appraised according to study design, sample size, endpoint definition, external validation, confounding, and contemporary applicability; no formal risk‐of‐bias or evidence‐grading framework was applied.

OpenAI ChatGPT and Codex were used to assist with grammar checking, formatting, and refinement of tables. All AI‐assisted material was reviewed and edited by the authors, and factual statements and citations were checked against the original sources.

## 3. Definitions, Incidence, and Clinical Interpretation

Reported RHF incidence after LVAD implantation varies widely because the definition, time window, and event components have changed. The 2008 INTERMACS definition was relatively narrow and combined hemodynamic abnormalities with RVAD use or more than 1 week of inhaled nitric oxide or inotropic support. The 2014 INTERMACS definition required evidence of raised right‐sided filling pressure together with clinical congestion or liver or kidney dysfunction and graded severity according to the support required [7]. The 2020 MCS‐ARC definition then separated early acute RHF, early postimplant RHF, and late RHF [8]. The 2024 ESC‐HFA consensus uses the MCS‐ARC definition in its clinical guidance rather than proposing a separate definition [4].

Common research endpoints do not always match these formal definitions. The EUROMACS‐RHF endpoint, for example, includes right‐sided mechanical support, continuous inotropic support for at least 14 days, or inhaled nitric oxide for at least 48 h [9]. Other studies use similar treatment‐based composites or add death from RHF within 30 days. These endpoints capture severe clinical events, but they also reflect local decisions about weaning drugs and starting mechanical support. Definitions based on congestion may miss hemodynamic abnormalities; in one study, the noninvasive INTERMACS criteria were 32% sensitive and 97% specific for elevated right atrial pressure [10]. Authors should report the definition, time window, and each component of a composite endpoint. Early RHF comprises MCS‐ARC early acute and early postimplant RHF unless the original study applied another definition (Table 1).

**Table 1:** Formal definitions and research endpoints for right heart failure after left ventricular assist device implantation.

Definition or research endpoint	Time window and operational criteria	Effect on reported incidence and study comparison	Clinical interpretation
Formal consensus definitions			
INTERMACS 2008 [7]	Persistent RV dysfunction with CVP > 18 mmHg and cardiac index < 2.0 L/min/m^2^; no left atrial pressure/PCWP > 18 mmHg, tamponade, ventricular arrhythmia, or pneumothorax; plus RVAD implantation or iNO/inotropic therapy for > 1 week.	A narrow hemodynamic and treatment‐dependent definition that generally yields lower incidence estimates. Comparisons with later definitions require endpoint harmonization.	Captures hemodynamically defined, treatment‐requiring RHF but can omit less overt or medically managed congestion.
INTERMACS 2014 [7, 10, 11]	Both required elevated CVP documented by RAP > 16 mmHg, dilated IVC without inspiratory variation, or JVP at least halfway up the neck; and peripheral edema > 2+, ascites/hepatomegaly, bilirubin > 2.0 mg/dL, or creatinine > 2.0 mg/dL. Severity is graded by treatment duration and RVAD use.	Adds physical examination, imaging, congestion, and end‐organ criteria and identifies a broader phenotype than the 2008 definition.	Captures clinical congestion, whereas the severity grade remains influenced by treatment duration and local practice.
MCS‐ARC 2020 [8]; used in ESC‐HFA 2024 [4]	Early acute: temporary or durable RVAD, including ECMO in the consensus nomenclature, before leaving the operating room. Early postimplant: RVAD within 30 days; failure to wean inotropes, vasopressors, or iNO within 14 days, or restart within 30 days for ≥ 14 days, with ≥ 2 clinical findings or ≥ 1 manifestation; death within 14 days while on support and meeting diagnostic criteria. Late: RVAD > 30 days or RHF hospitalization > 30 days requiring intravenous diuretics/inotropes for ≥ 72 h plus diagnostic criteria.	Separates immediate, early postimplant, and late events. Incidence changes according to the categories and treatment components included.	Current consensus framework for trials and registries. Planned and rescue RVAD use should be reported separately. ECMO counts toward the event definition but is not RV‐specific bypass support.
Commonly used research endpoints			
EUROMACS‐RHF endpoint [9]	Right‐sided mechanical support, continuous inotropes for ≥ 14 days, or iNO for ≥ 48 h within 30 days after surgery.	Counts prolonged drug or iNO treatment as an event; local weaning practice can change incidence and apparent model performance.	A treatment‐dependent endpoint developed for risk‐model derivation and validation; it differs from RVAD‐only and MCS‐ARC outcomes.
Severe early RHF composite in an INTERMACS analysis [12]	Death from RHF within 30 days, RVAD implantation within 30 days, or inotropic support beyond 14 days.	Broader than an RVAD‐only endpoint. In this analysis, the association was driven mainly by prolonged inotropic support.	The components differ in severity and susceptibility to local practice; report component results alongside the composite.

*Note:* Definitions and endpoints are abbreviated for comparison; formal adjudication should follow the original documents. In MCS‐ARC, ECMO is included within RVAD‐related event criteria but is not an RV‐specific bypass strategy. Composite outcomes should be reported with their individual components.

Abbreviations: CVP, central venous pressure; ECMO, extracorporeal membrane oxygenation; iNO, inhaled nitric oxide; INTERMACS, Interagency Registry for Mechanically Assisted Circulatory Support; IVC, inferior vena cava; JVP, jugular venous pressure; MCS‐ARC, Mechanical Circulatory Support Academic Research Consortium; PCWP, pulmonary capillary wedge pressure; RAP, right atrial pressure; RHF, right heart failure; RVAD, right ventricular assist device.

Registry data also show that RHF changes over time. Among 5537 continuous‐flow LVAD recipients, RHF was present in 24% at 1 month, whereas new RHF was first identified in 5.1% at 3 months and 4.8% at 6 months [11]. Persistent RHF was associated with worse survival than RHF that resolved. In a separate cohort of 2200 HeartMate 3 recipients, early RV failure occurred in 18.3% and 6.4% required RVAD support [3]. These estimates reflect different cohorts, devices, time windows, and definitions; incidence must therefore be interpreted with the definition and timing used.

Study‐specific composite outcomes should be reported together with their individual components. In one INTERMACS analysis, severe early RHF included death from RHF within 30 days, RVAD implantation within 30 days, or inotropic support for more than 14 days [12]. These components differ in clinical severity and are influenced to different degrees by local treatment practices. In that study, prolonged inotropic support accounted for much of the observed association. Therefore, a composite rate should not be interpreted as equivalent to either RVAD use alone or an outcome defined by contemporary consensus criteria.

## 4. Mechanisms of Early RHF

RV systolic performance depends on free‐wall contraction and ventricular interdependence through the interventricular septum and shared myocardial fibers [13, 14]. Changes in LV loading and septal geometry after LVAD implantation can therefore alter RV performance. Pressure‐volume studies in LVAD recipients have also demonstrated occult RV dysfunction and impaired RV–pulmonary arterial coupling that may not be apparent from conventional measures [15].

LVAD support changes RV loading in opposing directions. LV unloading lowers left‐sided filling pressures and can reduce pulmonary vascular load, thereby decreasing RV afterload. At the same time, increased systemic flow increases venous return and RV preload. LV decompression also alters septal geometry; marked leftward septal displacement may impair RV geometry and tricuspid leaflet coaptation, although the net effect of greater LV unloading on RV contractility is not uniform [14, 16, 17]. An RV with adequate reserve can accommodate the increase in preload, whereas limited reserve may lead to RV dilation, increased wall stress, and worsening functional tricuspid regurgitation.

Perioperative factors can further reduce RV reserve or increase pulmonary vascular load. Cardiopulmonary bypass, ischemia–reperfusion injury, bleeding and transfusion, inflammation, arrhythmia, hypoxemia, hypercapnia, acidosis, and excessive airway pressure may impair RV contractility or increase afterload. In a susceptible RV, these changes can precipitate or worsen RV–pulmonary arterial uncoupling. As RV output falls, LV filling and LVAD flow decline, systemic perfusion deteriorates, and reduced coronary perfusion pressure may further impair RV function, creating a reinforcing low‐output cycle depicted in Figure 1.

**Figure 1:**
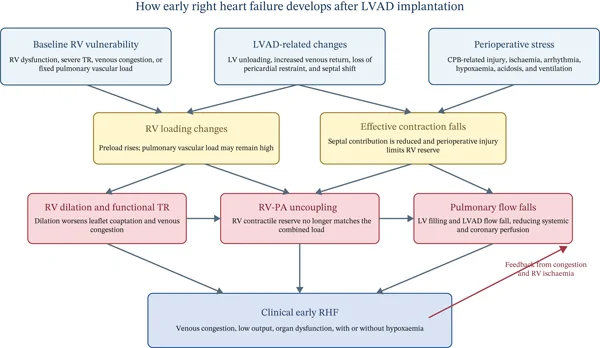
Main pathways leading to early right heart failure after left ventricular assist device implantation. Baseline RV vulnerability, the hemodynamic effects of LV unloading, and perioperative stress increase RV preload or afterload and reduce effective contraction. RV dilation, functional tricuspid regurgitation, and RV‐pulmonary arterial uncoupling reduce pulmonary flow, LV filling, and LVAD flow; venous congestion and impaired coronary or organ perfusion further worsen RV function. Arrows show the principal directions of interaction. *Source:* Original figure created by the authors from the evidence discussed in Section 4. CPB, cardiopulmonary bypass; LV, left ventricle; LVAD, left ventricular assist device; PA, pulmonary artery; RV, right ventricle; TR, tricuspid regurgitation.

## 5. Preimplant Assessment and Risk Prediction

### 5.1. Clinical Phenotype and Biomarkers

Preimplant risk is better characterized by a cluster of congestion, impaired RV function, clinical acuity, and limited end‐organ reserve than by any single laboratory value. Prior RHF, elevated jugular venous pressure, ascites or edema, severe tricuspid regurgitation, renal or hepatic dysfunction, multiple inotropes, temporary mechanical support, and INTERMACS Profiles 1–2 have all been associated with higher risk [4, 18, 19]. A 2026 meta‐analysis of 19 studies comprising 31,591 patients identified preoperative factors associated with subsequent RVAD requirement across clinical, hemodynamic, echocardiographic, and laboratory domains [20]. Many of these variables also reflect overall illness severity; preoperative mechanical support, for example, is affected by confounding by indication because its use reflects hemodynamic instability. These variables support optimization and contingency planning but do not establish isolated causal risk factors.

Natriuretic peptides and markers of hepatic, renal, nutritional, and coagulation status may add context to clinical assessment, but none are sufficiently RV‐specific for stand‐alone prediction. No biomarker threshold has been validated as an isolated criterion for LVAD candidacy or planned RV support.

### 5.2. Imaging

Echocardiography remains the first‐line imaging modality because it integrates RV size and systolic function, tricuspid regurgitation, septal geometry, and estimates of filling pressure. TAPSE and tricuspid annular systolic velocity are readily obtained but are load‐dependent and predominantly reflect longitudinal motion. Fractional area change and RV free‐wall longitudinal strain provide complementary measures of RV systolic performance; less negative RV free‐wall strain has been associated with post‐LVAD RHF, although acquisition methods and reported cutoffs vary [18, 19, 21–23]. A 2025 meta‐analysis of 39 studies involving 2975 patients found associations with larger RV end‐diastolic diameter and less negative RV free‐wall strain; TAPSE was also associated with RHF but showed substantial heterogeneity (*I* ^2^ = 86.1*%*) [24]. Thresholds derived from pooled data are constrained by differences in endpoints, patient acuity, and measurement methods. No single echocardiographic measure reliably rules early RHF in or out.

Three‐dimensional echocardiography, cardiac CT, and cardiac magnetic resonance provide additional information on RV geometry, volumes, and tissue characteristics [21, 25, 26]. Their incremental predictive value for early RHF remains limited, and their main role is to resolve inadequate conventional imaging or define anatomy relevant to surgical planning.

### 5.3. Invasive Hemodynamics and Functional Reserve

Right‐heart catheterization provides direct measures of congestion, forward flow, and pulmonary vascular load. A high right atrial pressure‐to‐pulmonary capillary wedge pressure ratio, low pulmonary artery pulsatility index (PAPi), low RV stroke work index, and increased pulmonary arterial elastance have each been associated with early RHF [27–29]. Reported thresholds vary across cohorts because measurements are influenced by loading conditions, vasoactive therapy, rhythm, measurement timing, and the RHF endpoint used.

Multicenter studies support dynamic hemodynamic response as a marker of RV reserve. In a 70‐patient retrospective study, higher peak stroke volume index during sodium nitroprusside infusion was associated with a lower risk of MCS‐ARC–defined early RHF, with an AUC of 0.78 [30]. A 2026 multicenter study of 160 LVAD recipients reported early RV failure in 58 patients (36.3%); a PAPi increase of < 2.2 during nitroprusside challenge was independently associated with RV failure (OR 4.56, 95% CI 1.88–11.07) [31]. These findings justify prospective validation of dynamic thresholds. Both analyses were retrospective and selected patients undergoing vasodilator testing, so hemodynamic results should be integrated with loading conditions, current therapy, and imaging.

### 5.4. Comparative Performance and Clinical Use of Risk Models

Risk models can structure preimplant assessment, but they were developed across different device eras, populations, and RHF endpoints (Table 2). The Michigan RV failure risk score largely reflects vasopressor dependence and end‐organ dysfunction and was derived in an era in which most devices were pulsatile‐flow [32]. The Kormos predictors were derived in 484 HeartMate II bridge‐to‐transplant recipients [29]. CRITT is simple to calculate, but its derivation cohort compared successful isolated LVAD support with the need for biventricular support rather than the broader contemporary RHF syndrome [35].

**Table 2:** Structured comparison of commonly used risk models for early right heart failure after LVAD implantation.

Model and derivation cohort	Main variables and predicted outcome	Reported discrimination	External testing and calibration	Current role and evidence gap
Michigan RV failure risk score [32] (*n* = 197; mainly pulsatile‐flow devices)	Vasopressor use, AST, bilirubin, and creatinine; composite of prolonged inotropes/iNO, RVAD, or discharge on inotropes	AUC 0.73 in derivation	*C* = 0.62 in 116 continuous‐flow recipients [33]; *C* = 0.606 in a 2026 single‐center comparison [34]; calibration not reported in these comparisons.	Captures shock and organ dysfunction. Its device era and treatment‐based outcome limit current application; outcome benefit from score‐guided care remains untested.
Kormos model [29] (*n* = 484; axial‐flow trial cohort)	RA/PCWP > 0.63, ventilator support, and BUN > 39 mg/dL; RVAD or prolonged/late inotropes	Association model; no widely used bedside probability estimate	*C* = 0.62 with an INTERMACS 3.0 outcome in one external cohort [33]; no calibrated bedside probability was tested.	Provides useful warning variables from a single‐device, mainly bridge‐to‐transplant cohort; it does not define a contemporary treatment threshold.
CRITT [35] (*n* = 218; 167 LVAD, 51 BiVAD)	CVP > 15 mmHg, severe RV dysfunction, intubation, severe TR, and tachycardia; need for biventricular support	Clear separation in the derivation cohort	*C* = 0.60 (95% CI 0.50–0.71) in 116 patients [33]; *C* = 0.653 in a 2026 single‐center comparison [34]; formal calibration not reported.	A practical screening score without calibrated prediction for contemporary post‐LVAD RHF.
EUROMACS‐RHF [9] (derivation *n* = 2000; validation *n* = 988)	Severe RV dysfunction, RA/PCWP ≥ 0.54, INTERMACS 1–3, ≥ 3 inotropes, and hemoglobin ≤ 10 g/dL; severe early RHF	*C* = 0.70 in derivation; 0.67 in internal validation	AUC 0.64 (95% CI 0.60–0.68) in 662 patients [36]; *C* = 0.670 with evidence of miscalibration in a 2026 single‐center study [34]; postoperative EUROMACS AUC 0.729 in a 105‐patient validation [37].	Discrimination and calibration vary across cohorts, and HeartMate 3 representation in the derivation cohort was limited. The postoperative formulation includes bypass time and is not a purely preoperative tool.
STOP‐RVF [38] (derivation, *n* = 798; external validation, *n* = 327)	Eleven clinical variables; RVAD or inotropes for > 14 days	*C* = 0.75 (95% CI 0.71–0.79)	*C* = 0.73 (95% CI 0.67–0.80); HeartMate 3 subgroup AUC 0.81 (95% CI 0.53–0.99); independent calibration evidence remains limited.	Interpretation is limited by the treatment‐dependent outcome and the imprecise HeartMate 3 estimate; the clinical benefit of score‐guided management remains untested.

*Note:* AUC and *C*‐statistics describe discrimination, not calibration or clinical benefit, and are not directly comparable across studies using different populations or RHF definitions. Calibration is stated only when reported.

Abbreviations: AST, aspartate aminotransferase; AUC, area under the curve; BiVAD, biventricular assist device; BUN, blood urea nitrogen; CI, confidence interval; CVP, central venous pressure; iNO, inhaled nitric oxide; PCWP, pulmonary capillary wedge pressure; RA, right atrial; RVAD, right ventricular assist device; TR, tricuspid regurgitation.

EUROMACS‐RHF achieved *C*‐indices of 0.70 and 0.67 in derivation and internal validation [9], whereas external validation in 662 patients yielded an AUC of 0.64 [36]. STOP‐RVF showed *C*‐statistics of 0.75 in derivation and 0.73 in external validation [38]. In its HeartMate 3 subgroup, the reported AUC was 0.81 with a wide confidence interval (0.53–0.99). These estimates do not support direct model ranking because study populations, endpoints, and analytic methods differed. In a 2026 single‐center comparison of 205 recipients, AUCs were 0.670 for EUROMACS, 0.653 for CRITT, 0.616 for Penn/Fitzpatrick, and 0.606 for Michigan; EUROMACS also showed evidence of miscalibration [34]. In another single‐center validation of 105 LVAD recipients, AUCs for established risk scores ranged from 0.394 to 0.729. Postoperative EUROMACS had the highest AUC, although it incorporates cardiopulmonary bypass time and is therefore not a purely preoperative model [37]. Calibration was not consistently reported for the other scores, and score‐guided treatment has not been shown to improve outcomes. Risk scores structure multidisciplinary assessment, optimization, and contingency planning; they do not define LVAD candidacy or mandate planned right ventricular support.

## 6. Early Postoperative Recognition

After implantation, the clinical problem shifts from estimating risk to recognizing evolving RHF and distinguishing it from other causes of postoperative low flow or shock. Recognition depends on changes over time in venous congestion, forward flow, end‐organ perfusion, RV imaging, and LVAD performance. Persistent or rising CVP, falling cardiac index or mixed venous oxygen saturation, increasing lactate, worsening renal or hepatic function, RV dilation or dysfunction, and reduced LVAD flow should raise concern when they persist despite initial optimization. Serial hemodynamic measures such as PAPi may provide additional context, whereas septal position should be interpreted together with LV filling, volume status, and pump speed [4, 8].

Alternative causes of low LVAD flow or postoperative shock—including hypovolemia, bleeding, tamponade, excessive pump speed or suction, and device inflow or outflow obstruction—should be distinguished from primary RV failure. Pulmonary embolism, pneumothorax, arrhythmia, respiratory failure, and sepsis may precipitate or aggravate RV dysfunction and should also be sought. Consensus thresholds classify RHF outcomes in studies; they are not validated triggers for mechanical‐support escalation [4, 8]. The direction of change after correction of reversible factors is often more informative than an isolated postoperative value.

## 7. Prevention and Management

### 7.1. Preload, Rhythm, Ventilation, Contractility, and LVAD Settings

Management should be directed at the dominant hemodynamic abnormality rather than follow a fixed drug sequence. Excessive RV preload increases wall stress and may worsen tricuspid regurgitation, whereas inadequate filling limits LVAD preload and pump flow. Persistent congestion should be treated with diuresis, with ultrafiltration or renal replacement therapy considered when volume overload is refractory or accompanied by renal indications. Empiric volume loading should be avoided; cautious fluid administration is appropriate when there is evidence of inadequate filling. Sinus rhythm and atrioventricular synchrony should be restored when possible. Hypoxemia, hypercapnia, acidosis, atelectasis, and excessive airway pressure can increase pulmonary vascular resistance and should be corrected. LVAD speed should be adjusted with echocardiographic and hemodynamic guidance to achieve adequate LV unloading without persistent leftward septal displacement or suction events [4].

Milrinone or dobutamine is commonly used when low output persists despite correction of loading conditions. Drug selection depends on systemic blood pressure, renal function, arrhythmia risk, and the desired balance between inotropy and pulmonary vasodilation; no adequately powered LVAD‐specific randomized trial has established superiority of one agent. Norepinephrine or vasopressin may be required when vasoplegia compromises systemic pressure and coronary perfusion. These practices are supported predominantly by physiological rationale, expert consensus, and observational experience rather than comparative LVAD‐specific trials [4].

### 7.2. Pulmonary Vasodilators

Inhaled nitric oxide and inhaled epoprostenol reduce pulmonary vascular load while limiting systemic vasodilation compared with intravenous pulmonary vasodilators. The INSPIRE‐FLO randomized equivalence trial included 231 patients undergoing LVAD implantation or heart transplantation and found similar rates of postoperative RV failure with inhaled epoprostenol and inhaled nitric oxide (25.0% vs. 22.5%) [39]. INSPIRE‐FLO establishes clinical equivalence between the two inhaled agents when selective pulmonary vasodilation is indicated; it did not test pulmonary vasodilation against placebo.

Routine preimplant phosphodiesterase‐5 inhibitor therapy is not supported as a strategy to prevent early RHF. An INTERMACS propensity‐matched analysis found a higher rate of severe early RHF with preimplant use, with substantial confounding from greater baseline pulmonary vascular disease [12]. Evidence for routine oral pulmonary vasodilator treatment after LVAD implantation also remains insufficient. Oral therapy should be reserved for a defined pulmonary vascular indication and reassessed as hemodynamics evolve. Table 3 compares the evidence supporting these strategies.

**Table 3:** Evidence basis and limitations of common prevention and treatment strategies.

Intervention	Evidence basis	Reasonable clinical conclusion	Main limitation
Hemodynamic optimization, ventilation, rhythm control, and LVAD speed adjustment	Physiology, consensus statements, and observational practice [4]	Basic care supported by physiology and consensus; serial echocardiography and invasive hemodynamics are reasonable when instability persists.	No standardized LVAD‐specific randomized protocol; most targets come from physiology, consensus, and clinical experience.
Inhaled epoprostenol versus inhaled nitric oxide	Double‐blind randomized equivalence trial; *n* = 231 advanced heart‐failure operations [39]	Either inhaled agent is reasonable when selective pulmonary vasodilation is indicated.	The active‐drug comparison included both transplant and LVAD procedures and did not test pulmonary vasodilation against placebo.
Routine preimplant PDE5 inhibitor	Large propensity‐matched registry study [12]	Do not use routinely only to prevent early RHF; reserve treatment for a defined hemodynamic indication.	Observational comparison affected by confounding by indication.
Concomitant tricuspid surgery	Randomized TVVAD trial with 12‐month follow‐up; *n* = 71 [40, 41]	Randomized data do not support routine surgery solely to prevent RHF; residual TR was reduced.	Small trial; effects in selected anatomical subgroups remain undefined.
Planned or early temporary RV support	Retrospective cohorts, including two recent HeartMate 3 studies with different results [42–44]	Prepare early for support in high‐risk patients; current data do not define a single best implantation time.	No randomized timing trial; treatment choice and timing reflect baseline severity and postoperative events.
Percutaneous versus surgical RVAD	Single‐center retrospective comparison; *n* = 40 [45]	Both approaches improved hemodynamics; survival comparison was underpowered.	Sample size and center‐specific selection limit mortality comparison.
Post‐LVAD transcatheter tricuspid repair	Case reports [46, 47]	Restrict use to carefully selected patients at experienced centers.	Evidence is limited to case reports; controlled LVAD comparisons are absent.
RV‐specific molecular or metabolic therapies	Preclinical and non‐LVAD studies	Priority area for translational and clinical investigation.	Clinical outcome evidence in post‐LVAD RHF is not yet available.

*Note:* The evidence basis column summarizes the principal study design(s) supporting each intervention. Evidence was appraised narratively according to study design, sample size, endpoint definition, reported limitations, and contemporary applicability; no formal risk‐of‐bias tool or evidence‐grading framework was applied.

Abbreviations: ECMO, extracorporeal membrane oxygenation; LVAD, left ventricular assist device; PDE5, phosphodiesterase Type 5; RHF, right heart failure; RVAD, right ventricular assist device; TR, tricuspid regurgitation; VA, venoarterial.

### 7.3. Tricuspid Regurgitation

Significant tricuspid regurgitation reflects adverse RV geometry and venous congestion, but correction of the valve does not necessarily restore RV myocardial function. In the randomized TVVAD interim analysis, concomitant tricuspid valve surgery reduced residual tricuspid regurgitation but did not reduce moderate or severe RHF at 6 months [40]. Longer follow‐up of 71 randomized patients similarly found no difference in moderate or severe RHF at 12 months between patients undergoing concomitant tricuspid surgery and those undergoing LVAD implantation alone (51.4% vs. 50.0%) [41]. By 12 months, tricuspid regurgitation severity also no longer differed significantly between groups because regurgitation improved over time in the no‐surgery arm.

These findings do not support routine concomitant tricuspid surgery solely to prevent RHF. The decision should instead consider valve anatomy, annular dilation, intracardiac leads, RV geometry and recovery potential, and the additional operative burden. Post‐LVAD transcatheter edge‐to‐edge repair has been described only in isolated reports [46, 47] and remains investigational.

### 7.4. Temporary Mechanical Support and the Role of VA‐ECMO

Temporary RV support is considered when severe RV failure persists after correction of reversible causes, optimization of LVAD settings, vasoactive or inotropic therapy, and selective pulmonary vasodilation when indicated. In the operating room, refractory early acute RHF may prevent separation from cardiopulmonary bypass. After implantation, escalation is driven by persistent venous congestion, inadequate forward flow, and worsening end‐organ perfusion. Rising central venous pressure, low cardiac index and LVAD flow, increasing lactate, progressive renal or hepatic dysfunction, and severe RV dilation or dysfunction demand prompt multidisciplinary reassessment [4, 8]. No single finding provides a universal implantation threshold.

Some observational cohorts have reported better outcomes when RV support was planned or instituted upfront, although findings have not been consistent across studies. Historical data found better outcomes with planned biventricular support than with delayed conversion after isolated LVAD implantation [42]. A later single‐center study of 64 HeartMate 3 recipients who required RVAD support reported lower 90‐day mortality with upfront than rescue support (33.3% vs. 67.9%; adjusted odds ratio 0.23, 95% CI 0.06–0.80) [43]. Because treatment was nonrandomized and the cohort included only RVAD recipients, this association supports early contingency planning rather than universal preoperative RVAD placement.

A 2025 single‐center cohort of 192 HeartMate 3 recipients included 51 patients who required temporary RVAD support. Delayed implantation was not associated with significantly higher 1‐year mortality than concomitant implantation (hazard ratio 1.41, 95% CI 0.53–3.74) [44]. Current studies therefore support early contingency planning and separate reporting of treatment intent and implantation timing; they do not establish universal superiority of planned, concomitant, or early support.

RV‐specific support bypasses the failing RV by draining the right atrium or vena cava and returning blood to the pulmonary artery. Percutaneous approaches allow rapid deployment and avoid repeat surgical access; internal‐jugular dual‐lumen cannulation can facilitate mobilization. Surgical RA‐to‐PA cannulation permits larger cannulae and flexible flow. An extracorporeal RA‐to‐PA circuit can include an oxygenator when gas exchange support is required [45, 48]. Both strategies improved hemodynamics in a 40‐patient comparison [45]; comparative survival and long‐term outcomes remain untested. Configuration should match required flow, gas‐exchange needs, vascular access, anticipated duration and mobility, interaction with LVAD filling, and local expertise.

VA‐ECMO provides immediate rescue when RV failure is accompanied by profound systemic instability, severe gas‐exchange failure, or impending cardiopulmonary collapse. Venous drainage unloads the right heart, whereas systemic arterial return increases aortic pressure and LV afterload. In an LVAD‐supported circulation, this interaction requires close attention to LV filling, LVAD flow, aortic valve opening, and pulmonary congestion. In a 32‐patient retrospective series from an earlier LVAD era, 29 patients (90.6%) were weaned from ECMO after RV recovery and in‐hospital mortality was 25% [49]. This series establishes feasibility; comparative effectiveness against RVAD remains undefined. When gas exchange is required within a selective RV bypass strategy, an oxygenator can be incorporated into an extracorporeal RA‐to‐PA RVAD circuit.

Device selection should answer five clinical questions: How severe is the loss of pulmonary forward flow; is venous congestion or organ dysfunction worsening; is pulmonary vascular load or gas‐exchange failure important; is LV filling and LVAD flow adequate; and is RV recovery likely within a reasonable time? The answers define the physiological target, required configuration, and expected duration of support (Table 4).

**Table 4:** Temporary right ventricular support and VA‐ECMO for refractory early right heart failure.

Support class and configuration	Flow path and main effect	Gas exchange	Principal indication	Main limitations
Temporary RV‐specific support				
Transvalvular microaxial RVAD	IVC/RA to PA; bypasses the RV and restores pulmonary flow and LVAD filling	No	Predominantly isolated RV forward‐flow failure with adequate lung function; rapid percutaneous deployment	Bleeding, vascular injury, hemolysis, valve injury, migration, and limited duration or mobility depending on access; evidence comes from small series.
Extracorporeal RA/central venous‐to‐PA RVAD	Right‐sided venous drainage to pump and PA return; direct RV bypass with adjustable flow	No, unless an oxygenator is added	Severe perioperative RV failure when higher or readily adjustable flow is needed; percutaneous dual‐lumen or surgical cannulation may be used	Cannulation, bleeding, infection, thrombosis, recirculation, and decannulation risks; comparative evidence is observational [45].
Oxygenated RA‐to‐PA RVAD	RA/central venous drainage to pump, membrane oxygenator, and PA return; RV bypass plus pulmonary gas exchange	Yes	RV forward‐flow failure with important hypoxemia or lung dysfunction when selective RV bypass remains appropriate	Greater circuit complexity and blood‐surface exposure; bleeding, thrombosis, recirculation, and oxygenator complications; evidence is limited [48].
Broader cardiopulmonary support				
VA‐ECMO	Central venous/RA drainage to pump, oxygenator, and systemic arterial return; supports systemic circulation and bypasses the heart and lungs	Yes	Circulatory collapse, combined circulatory and respiratory failure, or rapidly evolving shock requiring immediate broad rescue	Systemic arterial return increases LV afterload. LV filling, LVAD flow, aortic valve opening, and pulmonary congestion require close monitoring [49].

*Note:* Temporary RV‐specific support returns blood to the pulmonary artery and is distinct from VA‐ECMO, which returns oxygenated blood to the systemic arterial circulation. An oxygenated RA‐to‐PA RVAD combines selective RV bypass with extracorporeal gas exchange.

Abbreviations: IVC, inferior vena cava; LV, left ventricle; LVAD, left ventricular assist device; PA, pulmonary artery; RA, right atrium; RV, right ventricle; RVAD, right ventricular assist device; VA‐ECMO, venoarterial extracorporeal membrane oxygenation.

## 8. Prognosis

The prognostic significance of early RHF depends on its severity and persistence. Severe RHF requiring prolonged vasoactive or mechanical support is consistently associated with higher mortality [3, 28, 29, 50]. In the STS‐INTERMACS analysis, patients with persistent RHF at 3 months had a 2‐year survival of 57%, compared with 78% among those whose RHF had resolved by 3 months [11]. The course of RHF therefore provides prognostic information beyond the initial postoperative diagnosis. Late RHF is also associated with lower survival and a greater burden of adverse events [51]. Early improvement must be assessed over time rather than inferred from a single postoperative measurement.

## 9. Evidence Gaps and Research Priorities

To address the current limitations in literature, this section outlines the primary evidence gaps and future research priorities that must be addressed:

1. Use one definition and report its components. Prospective studies should use the MCS‐ARC time windows, report each part of a composite outcome, and separate congestion, low output, and organ dysfunction from treatment‐based criteria. In RV support studies, planned and rescue implantation should be reported separately.
2. Test risk models in current LVAD populations. Existing scores need external validation and recalibration in diverse cohorts. Studies should report calibration and clinical usefulness as well as discrimination, and should test whether serial imaging or hemodynamic stress testing improves decisions beyond resting measurements.
3. Compare temporary support strategies and timing. Multicenter studies should use consistent definitions of planned, concomitant, early, and rescue support. Comparisons should include percutaneous RVAD, surgical RVAD, oxygenated RVAD, and VA‐ECMO and should report survival, organ recovery, complications, mobility, and later clinical course.
4. Define when to escalate and how to judge recovery. Decisions should use changes over time in congestion, forward flow, lactate, organ function, RV imaging, LV filling, and LVAD performance. Successful recovery should be defined after support is removed, not by device removal alone.
5. Clarify the role of tricuspid intervention and RV‐specific treatment. Current randomized evidence does not support routine concomitant tricuspid surgery only to prevent RHF, but selected patients may still benefit. Post‐LVAD transcatheter repair and therapies aimed at RV metabolism, fibrosis, microvascular function, or RV‐pulmonary arterial coupling require prospective study.

## 10. Evidence Limitations

Variation in RHF definitions, device eras, case mix, and treatment practices limits direct quantitative comparison of incidence, prediction models, and temporary support strategies. Most evidence for management decisions comes from retrospective cohorts, consensus statements, and small case series; randomized comparisons remain a research priority. Some pivotal device trials and secondary analyses were industry‐sponsored or included industry‐affiliated authors; the MOMENTUM 3 program is one example [1, 3]. Such relationships should be considered together with study design, endpoint definition, comparator, and consistency with independent cohorts.

## 11. Conclusions

Early RHF after LVAD implantation results from limited RV reserve, changing loading conditions, ventricular interaction, and perioperative injury. The selected definition determines which events are counted and directly affects reported incidence and apparent risk‐model performance. Existing scores organize preimplant assessment but cannot replace clinical, imaging, and hemodynamic evaluation or define thresholds for candidacy and planned RV support.

When RV failure persists after reversible causes have been treated and LVAD support has been optimized, temporary support should match the main physiological problem. RV‐specific bypass restores pulmonary forward flow, an oxygenator adds gas exchange, and VA‐ECMO provides broader rescue for severe combined circulatory or respiratory failure. Current evidence supports transparent endpoint reporting, multimodal risk assessment, and early contingency planning. Direct comparative studies are needed to determine the optimal timing and configuration of support.

## Author Contributions

Qing Gao and Shujie Yan contributed equally to this work.

## Funding

No funding was received for this manuscript.

## Disclosure

Figure 1 and all tables are original.

## Ethics Statement

The authors have nothing to report.

## Conflicts of Interest

The authors declare no conflicts of interest.

## Supporting information


**Supporting Information** Additional supporting information can be found online in the Supporting Information section. The Supporting Information provides the PubMed/MEDLINE search strategy for this narrative review, including the complete search strings, search date, and record counts for each search focus (Table S1). It also describes the literature selection and prioritization approach.

## Data Availability

Data sharing is not applicable to this article as no datasets were generated or analyzed during the current study.
