# Supplementary material for: Early Right Heart Failure After Left Ventricular Assist Device Implantation: A Narrative Review of Definitions, Risk Prediction, and Management
Source: Cardiovasc Ther. 2026 Oct 27;2026:2611216. doi: 10.1155/cdr/2611216 (PMC13711371; doi:10.1155/cdr/2611216)
Supplement: Supplementary file 1 [file CDR-2026-2611216-s001.docx]

Supplementary Material

**Early Right Heart Failure After Left Ventricular Assist Device Implantation: A Narrative Review of Definitions, Risk Prediction, and Management**

# Supplementary Table S1. PubMed/MEDLINE search strategy

Searches were run in PubMed on 29 September 2026 and covered database inception to the search date. No language filter was applied. Record counts reflect PubMed on the search date.

| **Search focus** | **Full search string** | **Records** |
| --- | --- | --- |
| Core search | ("Heart-Assist Devices"[Mesh] OR "left ventricular assist device"[Title/Abstract] OR "left ventricular assist devices"[Title/Abstract] OR LVAD[Title/Abstract]) AND ("right heart failure"[Title/Abstract] OR "right ventricular failure"[Title/Abstract] OR "right ventricular dysfunction"[Title/Abstract] OR RHF[Title/Abstract] OR RVF[Title/Abstract]) AND ("1000/01/01"[Date - Publication] : "2026/09/29"[Date - Publication]) | 1,560 |
| Definitions and incidence | ("Heart-Assist Devices"[Mesh] OR "left ventricular assist device"[Title/Abstract] OR "left ventricular assist devices"[Title/Abstract] OR LVAD[Title/Abstract]) AND ("right heart failure"[Title/Abstract] OR "right ventricular failure"[Title/Abstract]) AND (definition[Title/Abstract] OR definitions[Title/Abstract] OR incidence[Title/Abstract] OR INTERMACS[Title/Abstract] OR "MCS-ARC"[Title/Abstract]) AND ("1000/01/01"[Date - Publication] : "2026/09/29"[Date - Publication]) | 307 |
| Risk prediction, imaging, and haemodynamics | ("Heart-Assist Devices"[Mesh] OR "left ventricular assist device"[Title/Abstract] OR "left ventricular assist devices"[Title/Abstract] OR LVAD[Title/Abstract]) AND ("right heart failure"[Title/Abstract] OR "right ventricular failure"[Title/Abstract] OR "right ventricular dysfunction"[Title/Abstract] OR RHF[Title/Abstract] OR RVF[Title/Abstract]) AND (predict*[Title/Abstract] OR "risk score"[Title/Abstract] OR echocardiograph*[Title/Abstract] OR hemodynamic*[Title/Abstract] OR haemodynamic*[Title/Abstract] OR "pulmonary artery pulsatility index"[Title/Abstract]) AND ("1000/01/01"[Date - Publication] : "2026/09/29"[Date - Publication]) | 740 |
| Management and mechanical support | ("Heart-Assist Devices"[Mesh] OR "left ventricular assist device"[Title/Abstract] OR "left ventricular assist devices"[Title/Abstract] OR LVAD[Title/Abstract]) AND ("right heart failure"[Title/Abstract] OR "right ventricular failure"[Title/Abstract] OR "right ventricular dysfunction"[Title/Abstract] OR RHF[Title/Abstract] OR RVF[Title/Abstract]) AND ("right ventricular assist device"[Title/Abstract] OR RVAD[Title/Abstract] OR "temporary mechanical circulatory support"[Title/Abstract] OR ECMO[Title/Abstract] OR "extracorporeal membrane oxygenation"[Title/Abstract] OR "tricuspid regurgitation"[Title/Abstract]) AND ("1000/01/01"[Date - Publication] : "2026/09/29"[Date - Publication]) | 533 |

# Selection and prioritisation

The review focused on adults receiving durable LVAD support and on definitions, incidence, mechanisms, risk prediction, diagnosis, prevention, temporary right ventricular support, VA-ECMO, prognosis, and research priorities. Consensus definitions and clinical consensus statements were used for terminology and clinical framing. Systematic reviews, randomized trials, multicentre cohorts, external validation studies, and studies in contemporary continuous-flow or HeartMate 3 populations were prioritised. Smaller observational studies and case reports were retained when they addressed emerging or infrequently studied interventions for which higher-level evidence was unavailable.

Reference lists of key consensus statements, systematic reviews, and primary studies were examined for additional relevant publications. Paediatric-only studies, studies of right heart failure unrelated to durable LVAD support, duplicate reports that did not add distinct outcomes, and studies outside the review scope were not retained. No protocol was registered, and no formal prespecified screening or exclusion process or dual-reviewer selection was used. Evidence was appraised narratively rather than with a formal risk-of-bias or grading instrument. The SANRA domains were used as a reporting aid, not as a systematic-review framework or numerical quality score.

**Update through September 2026**

The update identified a directly relevant external validation study published on 9 September 2026 (Sales et al., PLoS One; PMID 42715207; doi:10.1371/journal.pone.0357025). It was added to the risk-model section because it compared several established scores using a defined adult durable-LVAD cohort and reported discrimination and calibration. Other September records were not added when they were paediatric-only, small descriptive series without a comparator, or overlapped with evidence already represented in the manuscript without changing the clinical interpretation.
